# Occupation-related respiratory diseases among sanitary workers in the workplace: a systematic review and meta-analysis

**DOI:** 10.3389/fpubh.2024.1501768

**Published:** 2024-11-26

**Authors:** Sina Temesgen Tolera, Bezatu Alemu, Dechasa Adare Mengistu, Alemayehu Deressa

**Affiliations:** ^1^Haramaya University College of Health and Medical Sciences, Harar, Ethiopia; ^2^Addis Ababa University College of Health Science, Addis Ababa, Ethiopia

**Keywords:** infections, occupation, prevalence, respiratory, sanitary workers, workplace

## Abstract

**Introduction:**

Emerging evidence from both developed and developing countries indicates that occupation-related respiratory diseases (ORRD) among sanitary workers constitute a significant public health challenge. These are because of the working environment, where employees are more likely to be at risk in an unsafe workplace, especially sanitary workers. Therefore, the purpose of this systematic review and meta-analysis was to estimate the prevalence of ORRD among sanitary workers.

**Methods:**

The Preferred Reporting Items for Systematic Reviews and Meta-Analyses (PRISMA) flow diagram was used, and the Population, Intervention, Comparison, Outcomes, and Study (PICOS) framework was applied to structure the review questions. Studies published in English from 2000 to 2022 were searched in databases and through other methods. Boolean logic (AND, OR, NOT), Medical Subject Headings (MeSH), and keywords were used as follows: (Occupational “OR” Job “OR” Work) AND (Respiratory tract diseases “OR” Respiratory tract infections “OR” Respiratory tract symptoms “OR” Respiratory tract problems) AND (Solid waste collectors “OR” Sewage workers “OR” Street sweepers “OR” Waste treatment worker) AND Countries. Stata MP/17 software was used for data analysis. A random effects model and restricted maximum likelihood were applied. A generic precomputed effect size for the prevalence of ORRD was employed at a 95% confidence interval (CI:95%).

**Results:**

A total of 23 studies were included, four from industrialized countries (*n* = 4) and seven from developing countries (*n* = 7), out of an initial 123 studies. Among the 4,521 sanitary workers, 1990 (44%), 1,651 (37%), and 880 (19%) were SS, SWCs, and STWs, respectively. Globally, the pooled prevalence of ORRD among all SWs was 32.56% (95%CI: 25.78, 39.34%). Among these, high-income and low-income countries had a prevalence of 20% (95%CI: 18.08%, 0.21.96%) and 35.17% (95%CI: 27.48, 42.76%), respectively. In the SS and SWC groups, the prevalence was 36.41% (95%CI: 26.69%) and 31.28% (95%CI, 18.64, 43.92%), respectively.

**Conclusion:**

The current systematic review and meta-analysis found that ORRD were common among the SWs. Due to numerous risk factors, these illnesses are more prevalent in low-income countries than in industrialized ones. Therefore, to reduce these risks for these groups—especially for street sweepers in low-income countries—government policy changes and other preventive measures are required.

## Graphical abstract

A graphic abstract on the prevalence of ORRD among sanitary workers in the workplace was developed for this systematic review and meta-analysis at a global level.

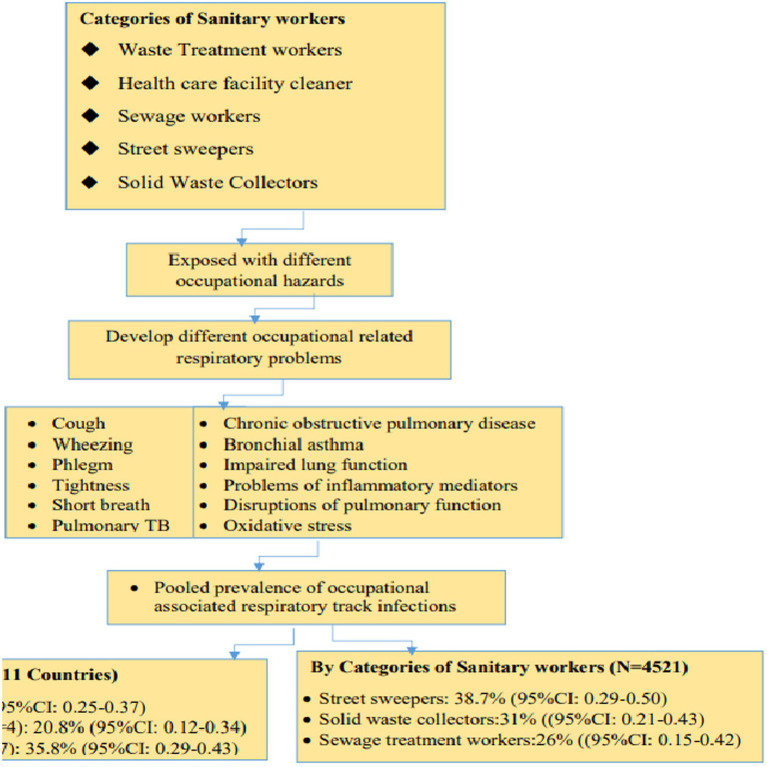

## Introduction

Sanitary workers, also known as sanitation workers, are responsible for maintaining and providing proper sanitation services in communities, such as homes, schools, hospitals, and other settings (1–4). However, millions of sanitation workers in developing countries are forced to work in hazardous conditions that violate their dignity and human rights and jeopardize their health and lives (3). They are often among the most marginalized groups, facing discrimination from others in society. They perform their duties without proper tools and lack legal protection (3), are economically disadvantaged (5), receive little attention in terms of occupational health and safety services, and are socially stigmatized (3) due to their working conditions.

Sanitation workers are exposed to various chemicals, dust, and aerosols, which can lead to respiratory ailments or other related health issues. This review examines the prevalence of respiratory infections, diseases, or conditions, as indicated by self-reported symptoms, clinically confirmed diagnoses, or recorded morbidity. These conditions include influenza-like symptoms, chronic obstructive pulmonary disease, upper airway inflation, coughing, phlegm production, wheezing, shortness of breath, nasal congestion, sore throat, headache, and asthma among the workers (6). These outcomes are more common in low- and middle-income countries compared to developed countries due to different risk factors (7). In these regions, sanitation workers, especially in low-income countries, may be exposed to bacteria, bacterial spores, or fungi through bioaerosols emitted from compost and waste (8). Moreover, they are often exposed to toxic materials, metal containers with residue chemicals, and heavy metals (9).

Several studies have identified age, educational status, type of home cooking fuel, prolonged working hours, employment status, past medical history, and use of face masks as factors associated with acute respiratory infections and respiratory symptoms (10–12). As another study also reported, inconvenience, ignorance, and the desire to save time were found to be the main reasons why sanitary personnel such as street sweepers did not use personal protective equipment. In addition, the training on the use of personal protective equipment (PPE) and occupational health and safety was not statistically significant in the current study. Respiratory problems were mainly caused by insufficient safety training, particularly on-the-job training, limited use of PPE during duty, and extended working hours. This is why it is expected that training will change workers’ perceptions of respiratory health issues and provide knowledge about how to safeguard their health in places where there is a high level of dust (13).

However, global statistics on respiratory illnesses linked to the sanitation industry are neither well-known nor accurately recorded. Therefore, although these issues are not yet fully recognized or assessed internationally, collated information about occupation-related respiratory diseases (ORRD) among sanitation workers is crucial for minimizing health risks among these groups worldwide. As a result, the authors posed four research questions (RQs_1–4_) to estimate the pooled prevalence of ORRD among SWs, which were included in the scope of this systematic review and meta-analysis. QR1: What is the prevalence of ORRD among SWs worldwide? QR2: What is the prevalence of ORRD among SWs in high-income and low-income countries? QR3: What is the pooled prevalence of ORRD among SWs from 2000 to 2015 and 2016 to 2022? RQ4: What is the pooled prevalence of ORRD among SWs after excluding the smallest and highest outcomes?

## Materials and methods

### Review protocol

The Preferred Reporting Items for Systematic Reviews and Meta-Analyses (PRISMA) protocol was used, and the flow diagram was modified from Page et al. (14). Meanwhile, the Population, Intervention, Comparison, Outcomes, and Study (PICOS) protocol was used for questions pertaining to this systematic review.

### Eligible criteria for review

The population (in this case, sanitary workers, using PICOS, the intervention, the comparison, the results, the kind of research design, the year of publication, the clarity of the articles using the PRISMA checklist), and the languages were the primary goals of the eligibility criteria. These criteria, detailed below, were used for both inclusion and exclusion.

#### Inclusion criteria

PICOS was used to determine the eligibility:

- Population (P): - This included sanitary workers, namely street sweeping crews, hospital cleaners, sewage workers, and those who process wastewater.
- Intervention (I): This included exposure in the workplace.
- Comparison (C): This was not relevant.
- Outcomes (O): This included respiratory diseases or infections associated with occupational or workplace exposure.
- Type Study Design (S): Only cross-sectional studies were included.
- Language: - Studies published in English with complete English texts and abstracts were included.
- Articles/Studies: - Studies with defined objectives and methodologies, quantitative results, and data on occupational risk factors that are freely available upon publication were all considered.
- Year of Publication: Studies published between 2000 and 2023 were included.
- Heterogeneity: Studies with heterogeneity (*I*^2^) less than 90% were eligible.

#### Exclusion criteria

- Population: Due to the nature of their jobs and other factors, office cleaners, hotel cleaners, and restaurant cleaners were not included in this study.
- Study design: Non-cross-sectional studies such as individual or cluster randomized controlled trials (RCTs). Quasi-RCTs, non-RCTs, historically controlled studies, interrupted time-series studies, case–control studies, and cohort studies are examples of non-randomized controlled studies (NRS).
- Outcomes: Studies addressing the prevalence of non-occupation-related injuries, mental health issues, and non-occupation-associated risk factors that may aggravate respiratory symptoms were excluded.
- Articles/papers: -Studies lacking specific aims, objectives, or methods, as well as those requesting patient-level data that were not publicly available at the time of publication, were not included.
- Language: - Non-English language studies were excluded.
- Publication year: - Studies published before the year 2000 were excluded.
- Heterogeneity: Studies with heterogeneity (*I*^2^) greater than 90% were excluded.

### Searched databases

The following databases were searched using EndNote: ST, BA, AD, and DA. Electronic databases such as PubMed, Medline, Embase, and Global Health were also used.

### Searched strategies

ST, BA, AD, and DA were used as search engines. Medical Subject Headings (MeSH) terms with Boolean logic operators (AND, OR) were applied individually or in combination to search online: The search strategy included the type of outcome, the type of population, and the location where the studies were conducted. Accordingly, the following search strategy was used: (Occupational “OR” Job “OR” Work respiratory related diseases) AND (Respiratory tract infections “OR” Respiratory tract symptoms) AND (Sanitary Workers “OR” Solid waste collectors “OR” Municipality solid waste collectors “OR” “OR” Sewage workers “OR” Street sweepers “OR” Waste treatment worker “OR” Sewage and waste treatment workers “OR” Street sweepers) AND (High-income “OR” Developed country “OR” Industrialized country “OR” Low-income country).

### Data screening

ST, BA DA, and AD contributed to the data screening process. Microsoft Excel was used to screen the search results for articles from the databases. Full copies of the articles, along with the titles and abstracts, were separated for further evaluation. Finally, the reference management programs EndNote 20 and Zotero were used to manage and remove duplicate articles.

### Data extraction

ST, BA DA, and AD contributed to these activities. An Excel spreadsheet was used to extract the data, which included the following information: the primary author, year of publication, reference number, countries, study design, and job categories for sanitation employees. In addition, this section also included the instruments used to assess respiratory tract symptoms, risk factors, and any mitigation actions taken or provided.

### Data analysis

ST, BA, DA, and AD contributed to the data analysis using Stata version MP/17. The analysis was conducted based on 23 studies. The effect size index was the event rate (prevalence). A forest plot random effects model (restricted maximum likelihood) was applied to estimate the pooled prevalence of RT infections, along with sub-analyses of countries, categories, and years, with a confidence interval of 95%. Moreover, meta-regression (random effects using the Hedges method) was performed to test the heterogeneity of the eligible studies. In this analysis, the I-square (*I*^2^) test was used to examine the reported prevalence for heterogeneity. Subgroup analyses were conducted between the type of sanitary workers, between low- and high-income countries, and between the time interval of 2000–2015 and 2016–2022. Moreover, sensitivity analysis was conducted after removing the studies with the smallest (*n* = 2) and largest (*n* = 2) prevalence values of respiratory diseases, using a *p*-value of 0.05 (CI: 95%). In addition, a visual funnel plot was used to detect publication bias, with a *p*-value of 0.05 (CI:95%).

### Data synthesis

ST, AD, DA, and BA contributed to the data synthesis and description based on the original articles, using texts, tables, figures, and forest plots. The studies on occupationally associated respiratory tract diseases, infections, or symptoms were collated, characterized, and synthesized based on the minimum and maximum prevalence, pooled prevalence among sanitary workers, and country of origin. In addition, the authors of this review synthesized the risk variables for respiratory tract issues and the recommendations for future research.

### Quality assessment and publication bias

ST, BA, DA, and AD contributed to the assessment of the quality of the articles. The Joanna Briggs Institute (JBI) critical appraisal checklist, designed for cross-sectional research, was used to evaluate all aspects of the eligible studies (15). This checklist includes nine items. The results for each item were categorized as follows: (1) Yes, (2) No, (3) Unclear, and (4) Not applicable. An article is considered to have a high publication risk if it receives 5 “Yes” answer scores out of 9, a medium risk if it receives 5–7 scores, and a low risk if it receives 8–9 scores (Supplementary Table 1). Moreover, a visual funnel forest plot was also used to identify publication bias, with a scatter plot generated at a *p*-value of 0.05 (CI = 95%).

## Results

### Selection of the studies

**Figure 1:**
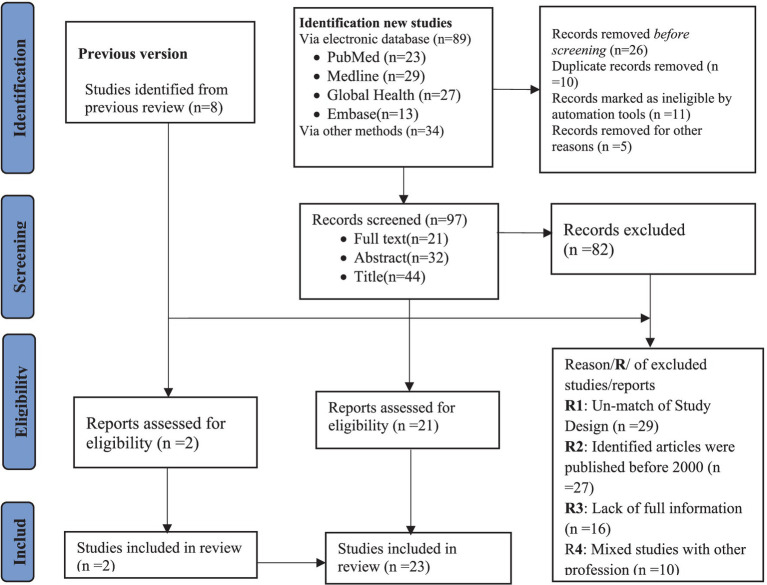
Flow diagram for the systematic review and meta-analysis, adopted from PRISMA 2020.

From the databases and other sources of collected data and reports, a total of 131 studies were identified. The next step involved screening 97 studies from the new identification and eight studies from the previous systematic review. After screening these records, a total of 82 studies were excluded, leaving 23 studies (*n* = 23) that were included in this systematic review and meta-analysis (Figure 1). The 23 included studies were conducted in 11 countries worldwide. Four of these studies were conducted in high-income countries such as Greece (16), Sweden (17), and the Netherlands (18, 19), while 19 studies were conducted in low-income countries. Among these, five studies were from Ethiopia (13, 20–22) and four studies from India (23–26). In addition, three studies were from Malaysia (27–29) and three from Nigeria (30–32). The remaining four studies were from Egypt (33), Sri lank (34), Tanzania (35), and South Africa (36).

### Study overview

Table 1 lists the authors, countries, study designs, methods used, classifications, risk factors for respiratory symptoms, and necessary measurements. Among the sanitary workers, the street sweepers were reported in some studies (13, 21, 24, 25, 30–33, 35), while the solid waste collectors were reported in other studies (20, 36, 37). The remaining populations consisted of sewage workers, as reported in some studies (18, 19, 26), with additional details attached as Supplementary Figure 2.

**Table 1:** Eligible studies included authors, countries, study designs, populations, risk factors, and measurements forwarded by the authors.

Reference	Year	Country	Design	Tool used f	Population (*N* = 4,521)	Risk factors	Action measures forwarded
(13)	2017	Ethiopia	CS	Questionnaires	SS (*n* = 405)	Lack of PPE Lack of training Lack of resting rooms Lack of showers	Forwarded interventions: Provide proper PPEs Provide right training Facilitate shower and resting rooms
(23)	2019	India	CS	Questionnaires	Sewage workers (*n* = 104)	Little attention to sewage workers Lack of full equipment	Forwarded interventions: Screening and public health interventions Provide sufficient PPE
(28)	2020	Malaysia	CS	Questionnaires	SWCs (*n* = 290)	Contact with pets and animals Underlying chronic diseases smoking Without education or only primary Lack of PPE Daily exposure to droplets	Forwarded interventions: Institutions and policymakers should understand the problems stemming from the occupation Provide PPE Ensure job rotation, morning and afternoon
(29)	2018	Malaysia	CS	Questionnaires	SWCs (*n* = 111)	Low compliance with PPE Direct contact with waste and prolonged exposure	Forwarded interventions: Compliance of workers with preventive measures is crucial Emphasis on further interventions to reduce exposure
(27)	2012	Malaysia	CS	Questionnaires	SWCs (*n* = 191)	Lower educational level Lack of training Insufficient equipment	Forwarded interventions: Provide proper education Proper training Full equipment
(34)	2017	Srilanka	CS	Questionnaires	SWCs (*n* = 41)	Lack of vaccination Lack of OHS training Lack of job and worker monitoring and evaluation	Forwarded interventions: Educate SWCs about the importance of vaccination programs. Encourage participation in health clinics.
(20)	2017	Ethiopia	CS	Questionnaires	SWCs (*n* = 546)	Not wearing a facemask on the job Have a history of past illnesses Sleeping disorders	Forwarded interventions: Wear facemasks Change working hours Those who have a history of past illnesses should get health information Encourage consultation for sufficient sleep
(16)	2021	Greece	Spirometric	Questionnaires	SWCs (*n* = 50)	Smoking cigarettes Lack of close follow-up Improper use of PPE	Smoking cessation Close follow-up Proper use of PPE
(24)	2014	India	CS/CG	Questionnaires	SS (*n* = 120)	Occupational exposures to dust without precautionary measures may predispose workers to respiratory symptoms.	Workers should use protective face masks, perform wet sweeping instead of dry sweeping during work, and use long-handled brooms
(35)	2012	Tanzania	CS	Questionnaires	SS (*n* = 102)	Inappropriate or poor-quality PPE Lack of follow-up on PPE utility Lack of medical intervention	Provide appropriate and high-quality PPE Frequent use of PPE Implement medical intervention (such as sputum testing, chest x-ray, and chest ultrasound)
(25)	2018	India	CS	Interview, Spirometer	SS(*n* = 80)	Not using protective masks while sweeping	Emphasize the importance of using protective masks
(21)	2021	Ethiopia	CS	Questionnaires	SS (*n* = 84)	Not using a nose/mouth mask while on duty and using coal/wood as cooking fuel are factors associated with acute respiratory infections among SSs.	The municipality should motivate and monitor workers’ use of PPE, including masks and gloves. Workers should use a mask while working. Workers should use clean cooking fuel at home.
(21)	2021	Ethiopia	CS	Questionnaires	SWCs (*n* = 84)	Not using a mask while on duty and using coal/wood as cooking fuel are factors associated with acute respiratory infections among SWCs.	The municipality should motivate and monitor workers’ use of PPE, including masks and gloves. Workers should use a mask while working. Workers should choose clean cooking fuel at home.
(33)	2015	Egypt	CS/CG	Questionnaires, spirometer	SS (*n* = 207)	Lack of regular checkups for RT issues Lack of OHS training Lack of PPE	Interventions forwarded: Supply adequate PPE Encourage regular medical checkups for RT issues
(32)	2020	Nigeria	CS	Questionnaires	SS (*n* = 250)	Low knowledge Lack of OHS training Improper use of PPE Lack of PPE	Recommended as: Raise awareness Proper training Ensure the availability and proper use of PPE
(31)	2005	Nigeria	CS	Pulse Dosimeter	SS (*n* = 200)	Lack of regular checkups for RT issues Lack of OHS training Lack of PPE Lack of face-washing facilities	Interventions Forwarded: Regular medical checkups Provide OHS training Provide sufficient PPE Hand- and face-washing stations should be encouraged to reduce contamination
(22)	2022	Ethiopia	CS	Questionnaires	SS (*n* = 392)	Educational status Working experience Past history of sinusitis Lack of PPE Lack of OHS training Lack of supervision	Interventions Forwarded: Recruiters should consider the socio-demographic background of workers during enrollment Provide sufficient PPE Provide OHS training. Ensure regular supervision
(26)	2017	India	CS	Questionnaires	SWC (*n* = 224); STW (*n* = 51)	Chronic bronchitis is highly associated with age, type of worker, gender, lack of PPE, tobacco use, health condition, and training	Interventions Forwarded: Socio-demographic factors of workers should be considered Inform workers with a past history of respiratory conditions Ensure the availability and proper use of PPE Provide OHS training
(18)	2001	The Netherlands	CS	Questionnaires and Endotoxin	Sewage workers (*n* = 147)	Lack of good hygiene practices Lack of PPE use	Promote good hygiene practices at the workplace to prevent some of these symptoms Ensure proper use of PPE
(19)	2005	The Netherlands	CS	Endotoxin Measurement	STW (*n* = 468)	Lack of modern technology to detect RT issues	The government should be aware of these problems to alleviate the risks for sanitary workers
(17)	2002	Sweden	CS	Standard Questionaries	STW (*n* = 257)	Daily exposure to sewage and liquid waste Lack and improper use of PPE	Clinical investigations are needed to determine the cause of reported symptoms among sewage workers, and further field studies are required
(36)	2021	South Africa	CS	Questionnaires, Obse.	SWCSs (*n* = 114)	Number of days worked, age, and infectious and chronic diseases	Conduct more awareness programs, provide training, ensure adequate PPE, and the provision of a mobile clinic at the landfill
(30)	2020	Nigeria	CS	Questionnaires	SS (*n* = 150)	working >8/day, living above the poverty line, low education level, and not being married	Health education programs focused on socio-demographic and ORRD issues should be held periodically

CS, Cross Sectional Study; CG, Group Control; COPD, chronic obstructive pulmonary disease; Obse., Observational checklist; PPE, Personal protective Equipment; OHS, Occupational Health and Safety; RD, Respiratory Disease/Infections/; SCWs, Solid Waste Collectors; SS, Street sweepers; STW, Sewage and Waste Treatment workers, Sewage, Sewage workers.

### Eligible countries

The current data were reviewed from eleven (11) countries worldwide. Of these countries, three were high-income countries (*n* = 4 studies) and seven were low-income countries (*n* = 19 studies). The top three reviewed studies were from Ethiopia (*n* = 5 studies), India (*n* = 4 studies), and Malaysia (*n* = 3 studies). In total, 23 studies were included (Supplementary Figure 1).

### Eligible population

Of the total eligible population (*N* = 4,521), 1990 (44%), 1,651 (37%), and 880 (19%) were street sweepers, municipal solid waste collectors, and sewage and waste treatment workers, respectively, listed in decreasing order (Supplementary Figure 2).

### Assessment tools

Of the 23 studies, 18 used standard questionnaires, three used questionnaires with spirometric measurement, and the remaining two used a pulse dosimeter and endotoxin measurement (Supplementary Figure 3).

### Pooled prevalence of ORRD by region

**Figure 2:**
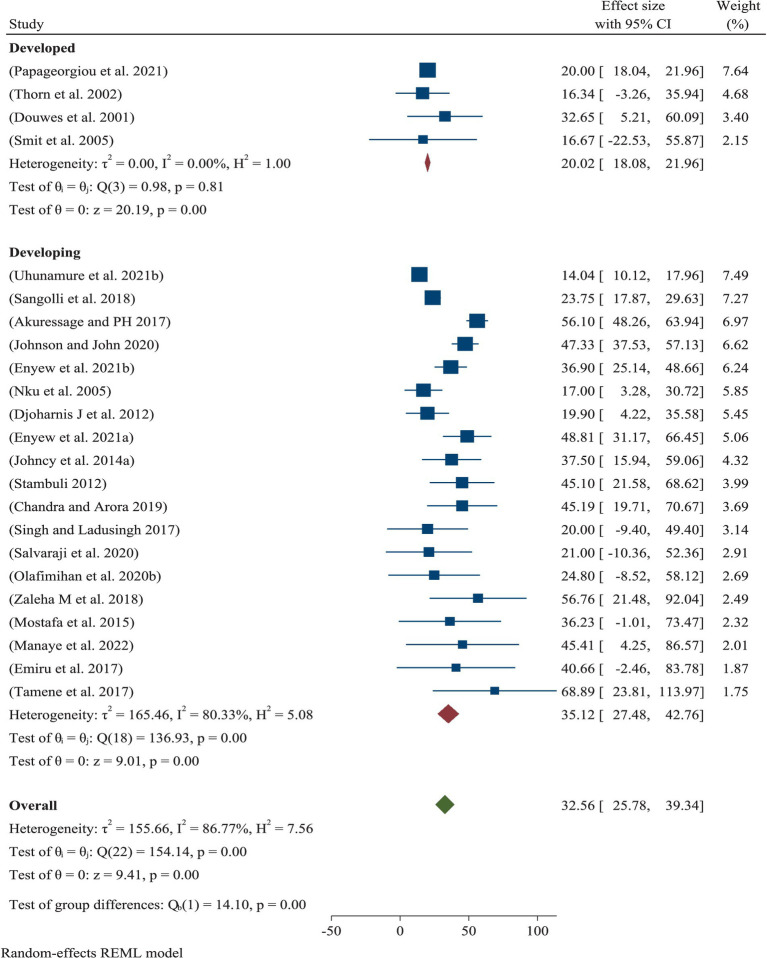
Pooled prevalence of ORRD among the sanitary workers by region and worldwide.

Across the world, the pooled prevalence of ORRD among all sanitary workers was 32.56% (95% CI: 25.78–39.34%), which is significantly associated with work-related conditions (Figure 2). Of this, 20% (95% CI: 18.08%–0.21.96%) and 35.17% (95% CI: 27.48–42.76%) were observed in high-income countries and low-income countries, respectively.

### Prevalence of ORRD by category

**Figure 3:**
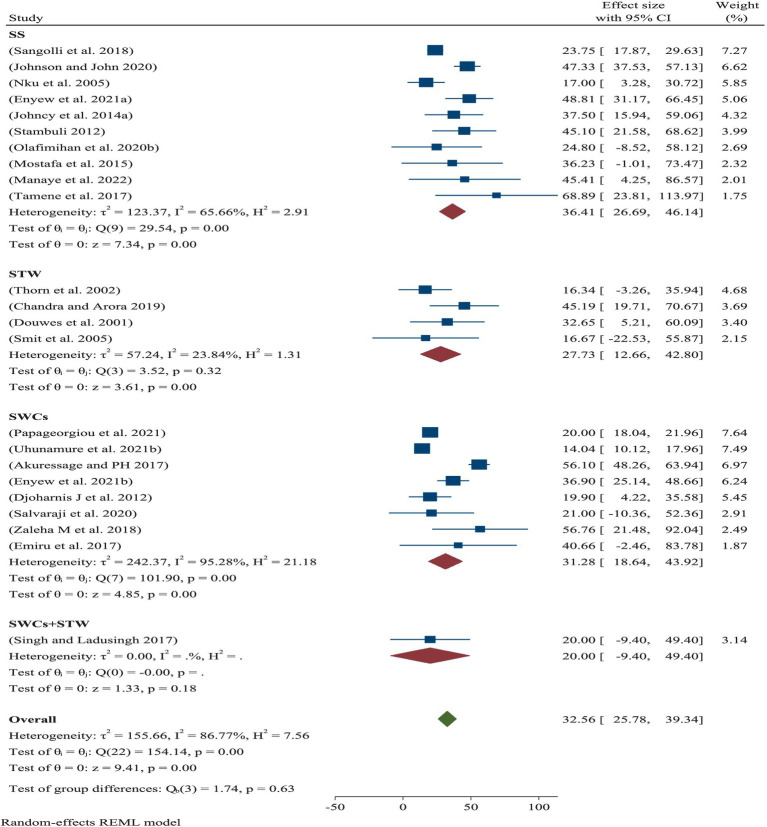
Pooled prevalence of occupational respiratory infections within a subgroup of sanitary workers.

Across the world, the pooled prevalence of ORRD among the street sweepers was 36.41% (95% CI: 26.69–46.14%), which is statistically significantly associated with work-related conditions. This was followed by solid waste collectors, with a prevalence of 31.28% (95% CI: 18.64–43.92%) (Figure 3).

### Prevalence of ORRD year-by-year

**Figure 4:**
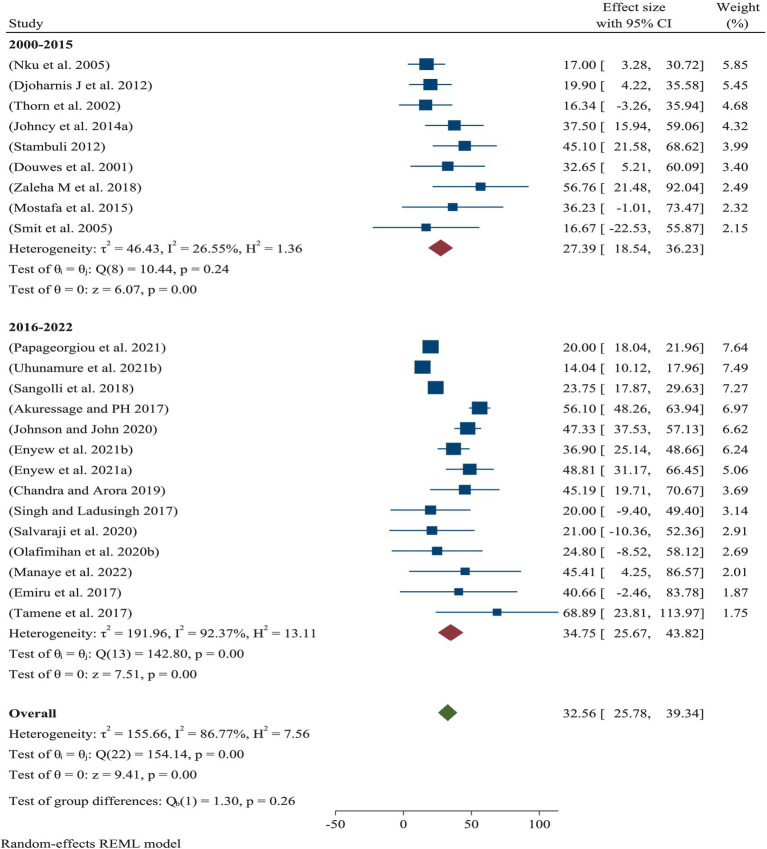
Prevalence of ORRD among the sanitary workers from 2000 to 2015 and 2016 to 2022.

Based on a year-by-year sub-analysis, the pooled prevalence of ORRD among the SWs was 27.39% (95% CI: 18.54–36.23%) from 2000 to 2015 and 34.75% (95% CI: 25.67–43.82%) from 2016 to 2022 (Figure 4).

### Sensitivity analysis

**Figure 5:**
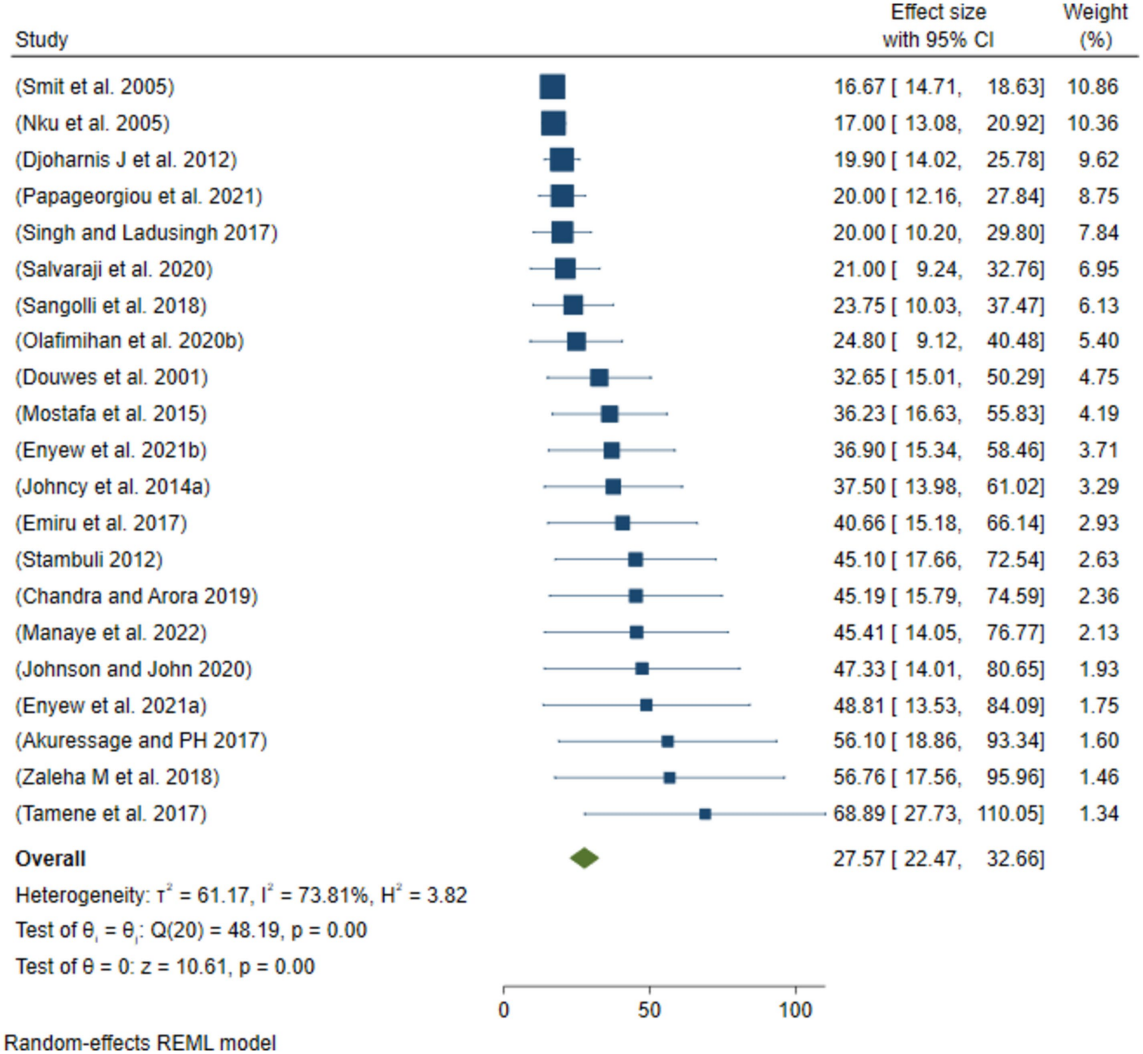
Sensitivity analysis by removing the two smallest outcomes of ORRD among the sanitary workers.

**Figure 6:**
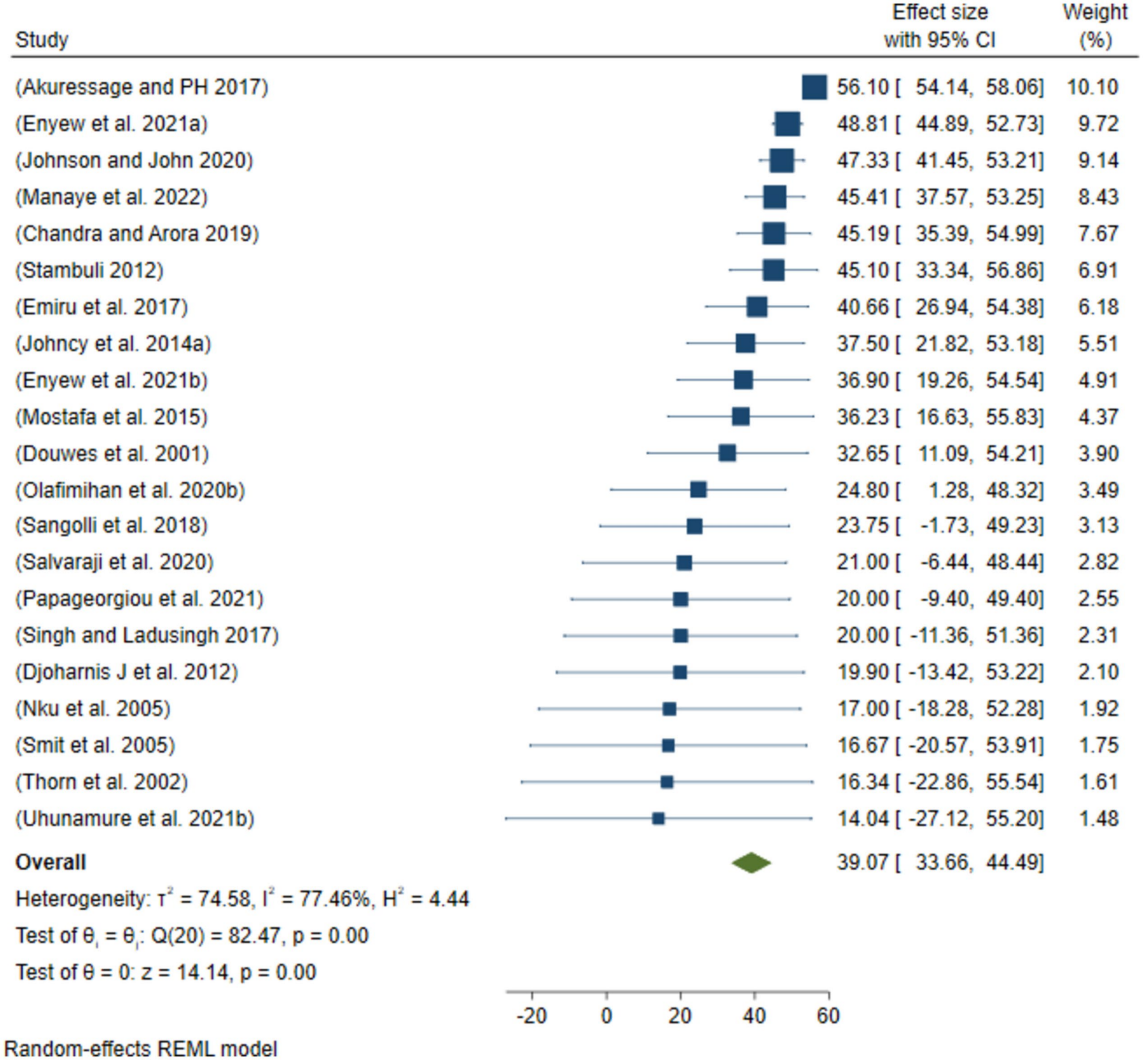
Sensitivity analysis by removing the two largest outcomes of ORRD among the sanitary workers.

After removing the two smallest outcomes (Figure 5) and two lowest/smallest “prevalence of respiratory track diseases” (Figure 6), the prevalence of ORRD 27.57% (95%CI: 22.47, 32.66%) among the sanitary workers worldwide was found to be 39.07% (95%CI: 33.66–44.49%) (Figure 5).

### Publication bias

**Figure 7:**
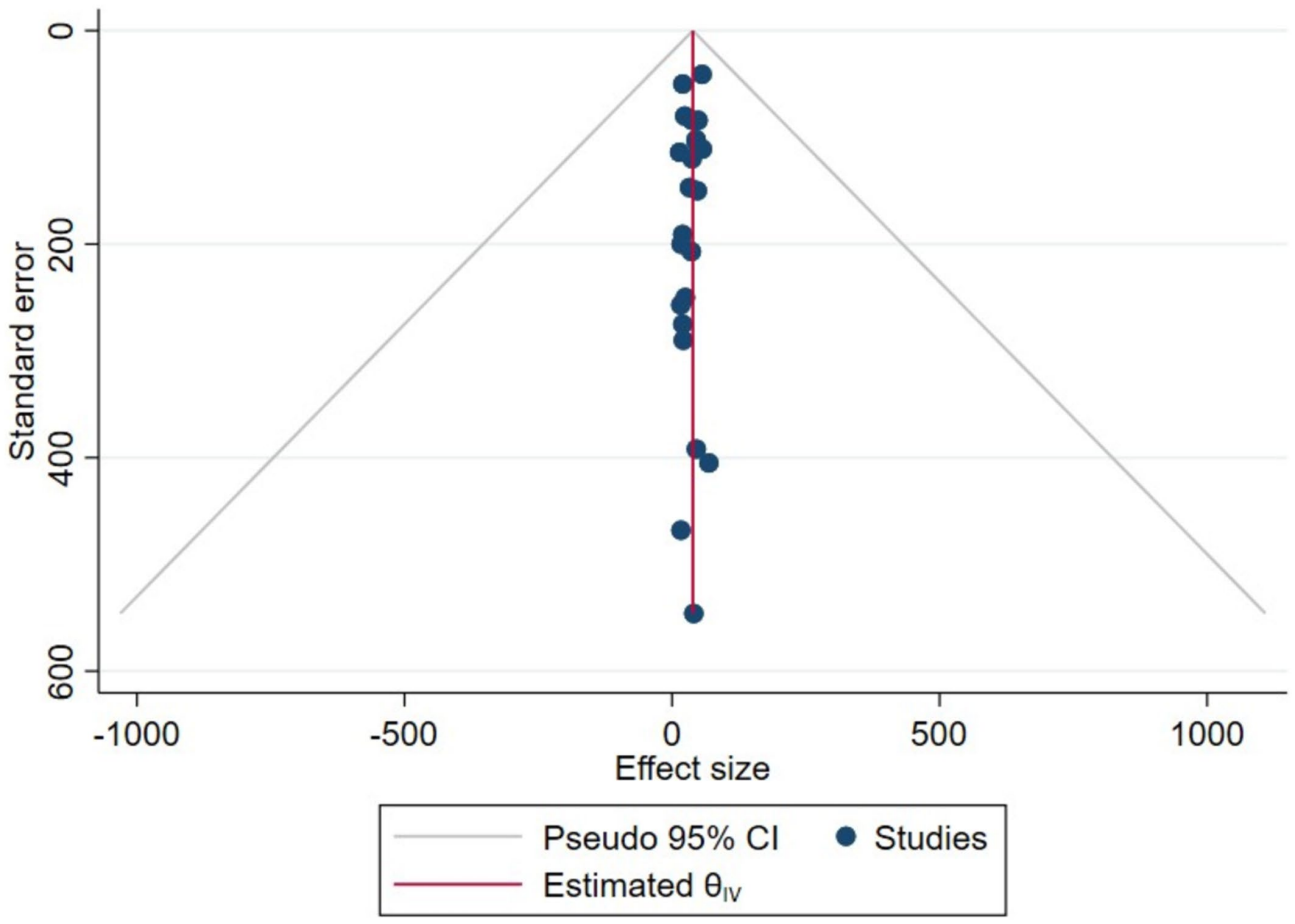
Plot funnel showing publication bias for the eligible studies, 2022.

According to the quantitative analysis using the JBI critical checklist, of 207 articles (100%), only 169 (81.64%) articles met the criteria for eligible studies (Supplementary Table 3). The scatter plots, which are asymmetrical and show all of the scatters pointing away from the vertical line and the funnel center, show the results of a thorough analysis of the funnel plot data (Figure 7).

## Discussion

The current systematic review and meta-analysis found that ORRD were the most common among SWs. These diseases included cough, wheezing, phlegm, tightness, shortness of breath, pulmonary tuberculosis, chronic obstructive pulmonary disease, bronchial asthma, impaired lung function, problems with inflammatory mediators, and disruptions in pulmonary functions (Table 1). Regarding the pooled prevalence, it was 32.56% (Figure 2), which is statistically significant for the occurrence of ORRD. The minimum prevalence of ORRD among the SWs was 14. %, which was found in South Africa, while the maximum prevalence was 56.1%, which was found in Malaysia (Figure 2). The disparity may be due to the study conducted in South Africa, which associated the prevalence with other work-related outcomes, such as musculoskeletal diseases, gastrointestinal disorders, and occupational injuries. As a result, the respondents might have paid less attention to the researcher or researchers. In reality, both were prevalent among municipal solid waste collectors in low-income countries, where there was little awareness, risky practices, extensive exposure to dust and chemicals, and a lack of respiratory protective equipment.

In the current review, the common risk factors for ORRD identified were lack of screening, daily contact with waste, little attention, low knowledge, and lack of safety training, all of which were correlated with the development of ORRD among the SWs (17, 23, 29, 32). In addition, lower educational status, work experience, living below the poverty line, low education level, not being married, working more than 8 h/day, and age were also the statistically significant risk factors for ORRD (22, 27, 30). Furthermore, lack of PPE, supervision, medical checks, and occupational health and safety training were identified as additional significant risk factors (13, 22, 23, 26, 30, 31, 33, 35). Regarding the sanitary industry, those with low compliance with PPE usage had a higher prevalence of ORRD (13, 20, 22, 23, 26, 29, 30). Other similar studies showed that failure to use a nose or mouth mask while on duty and using coal or wood as cooking fuel were also the risk factors. On the other hand, work conditions can extend to behavioral and economic factors, such as education, poverty, or healthcare access, which have been proven to be important determinants of ORRD (16, 26, 31).

Based on regional sub-group analysis, the pooled prevalence of ORRD among the SWs in high-income and low-income countries was 20 and 35.17%, respectively, both of which were statistically significant in relation to working conditions (Figure 2). This variation may be due to environmental and demographic factors that impact the diversity, composition, antimicrobial resistance, and presence of pathogenic microorganisms in the urban microbiome (38). A study found that the environmental microbiome may play a role in shaping respiratory diseases, serving as a potential mechanism for the development of respiratory issues among sanitation workers (39). The above disparity between high-income and low-income countries could be explained by the fact that due to extremely high microbial diversity and geographic variation, different health-associated species/genera are detected in different regions, as reported by Ful et al. (39) report. Another possible explanation could be that the majority of the studies focused on street sweepers, with a high prevalence found in low-income countries. This suggests that SWs in low-income countries may have been more exposed to dust, fog, smog, chemicals, and mist than their counterparts in high-income countries due to improper and insufficient PPE, lack of help, and lack of job and worker monitoring. In general, this finding shows that the severity of the problem may vary between developed and developing countries.

Subgroup analysis of the eligible populations revealed that street sweepers are potentially exposed to a range of occupational hazards, such as aerosols, dusts, fumes, and mists, all of which can lead to health problems such as respiratory illnesses. For example, bioaerosols present in cannabis-related work environments highlight the diversity, distribution, and abundance of these airborne particles, as well as the potential respiratory hazards they pose to workers (40). In the current meta-analysis, the pooled prevalence of ORRD among the street sweepers worldwide was 36.41%, which is statistically significant in relation to sanitary workers’ working conditions. Under these conditions, the minimum prevalence of ORRD was 17.0%, while the maximum was 68.9% (Figure 3). Similarly, the pooled prevalence of ORRD diseases among the municipality solid waste collectors was 31.28%, with a minimum and maximum prevalence of 14.0 and 56.8%, respectively. The lowest prevalence was found among the sewage workers and waste treatment workers, which was 27.73%. Moreover, based on the subgroup analysis by year intervals, the prevalence was 27.39% from 2000 to 2015 and 34.75% from 2016 to 2022 (Figure 4). This suggests that the pooled prevalence of ORRD has increased over time, which may be due to urbanization in low- and middle-income countries, leading to a greater need for sanitary workers, particularly street sweepers, in urban settings. Furthermore, sensitivity analysis was another key aspect of this systematic review and meta-analysis. The aim was to assess how the highest and the lowest values of ORRD could be affected under specific assumptions. Accordingly, after excluding the two extreme lowest disease outcomes, the pooled prevalence of ORRD among the sanitary workers worldwide was 27.57% (Figure 5). Similarly, after removing the two extreme largest outcomes, the pooled prevalence of ORRD among the SWs worldwide was 39.07% (Figure 6). This data indicate that there is a difference between the initial pooled prevalence of ORRD and the prevalence after eliminating extreme values, which may contribute to publication bias.

Regarding risk mitigation for ORRD among the SWs, the review identified that access to appropriate PPE and participation in training interventions are key recommendations (13, 22, 23, 26, 30, 31, 33, 35). For further management, routine medical interventions such as sputum tests, chest X-rays, and chest ultrasounds are suggested for risk mitigation. The review also found that SWs’ compliance with PPE as a preventive measure is crucial, with an emphasis on further interventions to reduce exposure during their daily work. Therefore, recruiters should consider the socio-demographic background of SWs when enrolling them, as well as their working hours per day. In addition, factors such as contact with pets and animals, daily exposure to contaminated droplets, and underlying chronic diseases are associated with the risk of ORRD (16, 24, 26, 28). The majority of the studies included in this review highlight the urgent need for screening, public health interventions, access to PPE, and creating awareness regarding workplace risks that can lead to ORRD (16, 24, 26, 28). In addition, institutions should provide rest rooms, shower rooms, and face- and hand-washing stations as these are expected to reduce and may even prevent the negative effects of inadequate rest spaces, lack of showers, and poor hygiene practices—factors that have been significantly associated with ORRD, which were reported in previous studies (18, 31).

Regarding the variations in the eligible studies and residual values, meta-regression was performed to assess the heterogeneity of the studies using the measurement methods of *I*^2^ (*I* squared), *T*^2^ (Tua squared), and *H*^2^ (H squared). Accordingly, the heterogeneity (*I*^2^) of the studies was 80.33%, falling within the range of 75 and 100% based on Higgins’ (41) cutpoint. This percentage indicates significant heterogeneity, suggesting unaccounted variability owing to residual heterogeneity in this review. Despite the fact that the high heterogeneity (*I*^2^ > 80%) observed suggests substantial variability among the studies—potentially due to differences in methodologies, geographic locations, and working conditions—it was managed by using a random effects model, conducting subgroup analysis, and performing meta-regression to explore and reduce heterogeneity. In addition, the heterogeneity among the studies, as measured by tau squared (*T*^2^), was 155.56, indicating that the absolute value of the true variance (heterogeneity) was 155.56. Furthermore, the *H*^2^ value (11.43) suggests that perfect homogeneity was not achieved, as it is higher than the ideal value of *H*^2^ (i.e., 1), according to Higgins’ (41) interpretation.

On the other hand, the overall quality of the articles included in this systematic review and meta-analysis was evaluated using the JBI critical evaluation criteria (Supplementary Table 1). According to these appraisal criteria, 65.3% (15/23) of the studies had low publication bias, while 34.7% had medium publication bias (Table 1). Therefore, according to the review, more than half of the qualified papers showed low publication bias. Furthermore, the statistical evidence derived from the funnel plot demonstrated that the scatter points were separated from one another and the vertical line of the funnel (Figure 7). This means that there was bias due to chance and inadequate methodological quality in the smaller studies, with selection bias being a major issue in this review. Some of these are the method used to sample study participants appropriately, the sample frame used to address the target population, and the challenges in applying reliable approaches to identify the condition. The majority of the studies did not specify the inclusion and exclusion criteria, as well as the process of selecting individuals responsible for workplace cleanliness (Supplementary Table 2).

## Strengths and limitations

### Strengths

Many of the qualifying studies met the required study design, total population size, and other criteria, which made it easy for us to incorporate the data into the programs and meet our deadlines. Furthermore, investigations on the prevalence of respiratory tract infections among various types of sanitary workers were classified in such a way that it was obvious that they were caused by ORRD, resulting in a straightforward search strategy.

### Limitations

This systematic review and meta-analysis has some limitations. First, many eligible studies in this review relied on self-reported data, which might have introduced recall bias. Second, almost all eligible studies used cross-sectional study designs, which made it difficult to establish causality for ORRD among the sanitary staff. Therefore, future studies should focus on longitudinal designs. Third, a significant amount of information in this review came from studies on street sweepers regarding ORRD among sanitary workers, which might have led to an unequal distribution of studies across other groups of sanitary workers. Fourth, a limitation is the scope of research and scientific rigor. The available research might not provide a sufficient foundation for policy recommendations or accurate estimates of disease burden due to its limitations. Finally, the current review was conducted at a global level, and as a result, the risk factors for occupation-related outcomes might have shown more variations, highlighting the need for issuing work health policies.

## Conclusion

The current meta-analysis revealed a high prevalence of ORRD among sanitary workers globally. The review also found that the prevalence was higher among sanitation workers in low-income countries compared to those in high-income countries. This disparity could be attributed to factors such as inadequate PPE, lack of screening, insufficient occupational health and safety procedures, lack of supervision and training, lack of commitment, and lack of focus on workplace risk by sector leaders. Therefore, this systematic review and meta-analysis recommends that it is very important to implement routine measures, such as providing access to protective equipment, offering occupational health and safety training, and ensuring regular supervision, to alleviate the high prevalence of ORRD among sanitary workers, particularly in low-income countries.

## What is already known about this topic?

Nowadays, the prevalence of occupation-related respiratory symptoms is increasing among all employees and workers, particularly sanitary workers, as a result of their working conditions, which are unsafe and unsanitary. These workers are often exposed to large amounts of waste in various work setups, such as municipalities, factories, commercial sectors, healthcare facilities, and plants. Furthermore, several studies have shown that sanitary workers are exposed to a variety of occupational hazards and accidents. They are also discriminated against, violated, and ignored by society. However, only a few studies have been conducted on quantifying ORRD among these groups, which is why we conducted a systematic review and meta-analysis across the globe.

## What does this study add?

This systematic review and meta-analysis provide evidence of the magnitude of occupation-related injuries among sanitary workers worldwide, an issue that has not been adequately reported. As a result, this review provides a brief overview of the global prevalence of these injuries and the associated work environments.

## How would this study affect research, practice, and/or policy?

The current report recommends that workplace guidelines on health issues for these groups be incorporated into occupational health and safety policies, laws, and amendments enacted by government authorities, especially the Ministry of Social Affairs, the Ministry of Health, and other organizations. The changes then need to be put into effect across all industries that provide employment, with close monitoring and enforcement.

## Data Availability

The original contributions presented in the study are included in the article/Supplementary material, further inquiries can be directed to the corresponding author.
