# Supplementary material for: Occupation-related respiratory diseases among sanitary workers in the workplace: a systematic review and meta-analysis
Source: Front Public Health. 2024 Nov 26;12:1501768. doi: 10.3389/fpubh.2024.1501768 (PMC11628511; doi:10.3389/fpubh.2024.1501768)
Supplement: Supplementary file 1 [file Data_Sheet_1.docx]

## **Eligible Countries**

Data were reviewed from eleven (11) countries across the world. Of these countries, 4 countries were from developed countries (n=4 studies) and 7 of them were from developing countries (n=19 studies). The first three leading reviewed studies found from Ethiopia (n=5 studies), India (n=4 studies), and Malaysia (n=3 studies). Totally, about twenty three studies were found from these countries (Sup. Figure 1).

Sup. Figure 1 Number of studies found from developed countries and developing countries, 2022

## **Eligible Population**

From total population of general sanitary workers (N=4521), 1990 (44%), 1651(37%) 880(19%) were street sweepers, Solid waste collectors/SWCs/ and sewage and waste treatment workers, respectively (Sup. Figure 2).

Sup. Figure 2 Categories of sanitary workers exposed OHS problems reviewed 2000-2021

## **Assessment tool**

From twenty three studies, about 18 studies were used standard questionnaires, 3 studies used Questionnaires with Spiro metric measurement. The rest 2 studies were used pulse dosimeter and Endotoxin measurement (Sup. Figure 3)

Sup. Figure 3 Assessment tool used by reviewed studies

## **Publication Bias**

The prevalence of occupational related respiratory symptoms or disease was evaluated using JBI Critical Appraisal Checklist for studies reporting prevalence data (Sup. Table 1)

JBI Critical Appraisal Checklist for studies reporting prevalence data

Reviewer______________________________________ Date_______________________________

Author_______________________________________ Year_________ Record Number_________

Sup. Table 1 JBI Critical Appraisal Checklist for studies reporting prevalence data

|  | Yes | No | Unclear | NA |
| --- | --- | --- | --- | --- |
| 1. Was the sample frame appropriate to address the target population? | □ | □ | □ | □ |
| 1. Were study participants sampled in an appropriate way? | □ | □ | □ | □ |
| 1. Was the sample size adequate? | □ | □ | □ | □ |
| 1. Were the study subjects and the setting described in detail? | □ | □ | □ | □ |
| 1. Was the data analysis conducted with sufficient coverage of the identified sample? | □ | □ | □ | □ |
| 1. Were valid methods used for the identification of the condition? | □ | □ | □ | □ |
| 1. Was the condition measured in a standard, reliable way for all participants? | □ | □ | □ | □ |
| 1. Was there appropriate statistical analysis? | □ | □ | □ | □ |
| 1. Was the response rate adequate, and if not, was the low response rate managed appropriately? | □ | □ | □ | □ |

Overall appraisal: Include □ Exclude □ Seek further info □

Comments (Including reason for exclusion)

________________________________________________________________________________________________________________________________________________________________________________________________

Based on above checklist, we evaluated each study and summarized as

**Publication Bias**

Twenty three studies included in this meta-analysis, which were evaluated based on JBI criteria that have nine statement. More than half percent are 65.3% (15/23) low publication bias, then followed by low publication bias (34.7%) (Table 1). For overall quality of the papers from 207 (100% quality of the paper), only about 169/207 (81.64%) fulfilled the JBI criteria (Sup. Table 2).

Sup. Table 2 Overall studies result by nine statement of JBI

| **Statement of JBI for Identified Studies (n=23)** | **Total Yes (Yes =X/23)** | **%** |
| --- | --- | --- |
| 1. Was the sample frame appropriate to address the target population? | 17 | 73.91% |
| 1. Were study participants sampled in an appropriate way? | 12 | 52.17% |
| 1. Was the sample size adequate? | 22 | 95.65% |
| 1. Were the study subjects and the setting described in detail? | 21 | 91.30% |
| 1. Was the data analysis conducted with sufficient coverage of the identified sample? | 19 | 82.61% |
| 1. Were valid methods used for the identification of the condition? | 18 | 78.26% |
| 1. Was the condition measured in a standard, reliable way for all participants? | 20 | 86.96% |
| 1. Was there appropriate statistical analysis? | 19 | 82.61% |
| 1. Was the response rate adequate, and if not, was the low response rate managed appropriately? | 21 | 91.30% |
| **Overall evaluation** | 169 | 81.64% |
